# Water usability as a descriptive parameter of thermodynamic properties and water mobility in food solids

**DOI:** 10.1038/s41538-023-00207-0

**Published:** 2023-06-14

**Authors:** Tingting Cui, Xukai Wu, Tian Mou, Fanghui Fan

**Affiliations:** 1 Department of Food Science and Engineering, College of Chemistry and Environmental Engineering, Shenzhen University, Shenzhen, Guangdong China; 2 Shenzhen Key Laboratory of Food Macromolecules Science and Processing, Shenzhen University, Shenzhen, Guangdong China; 3 School of Biomedical Engineering, Health Science Centre, Shenzhen University, Shenzhen, Guangdong China

**Keywords:** Biophysical chemistry, Biomaterials

## Abstract

A classic problem in preservation is the microbes can grow in low-moisture foods. In this paper, the water sorption, and thermodynamic properties of glucose/WPI solid matrices were measured, while their molecular mobility was analyzed and associated with the microbial growth of *D. Hansenii* at various *a*_*w*_ and 30 °C. Although the sorption isotherms, *T*_*g*_, and relaxation processes of studied matrices were affected by *a*_*w*_ and WPI, the microbial growth showed highly dependent on water mobility rather than *a*_*w*_. Hence, we introduced water usability (*U*_*w*_), derived from the mobility difference between system-involved water and liquid pure water explicating from the classical thermodynamic viewpoint, to describe the dynamic changes of water mobility in glucose/WPI matrices. Despite to *a*_*w*_, the yeast growth rate was enhanced at high *U*_*w*_ matrices concomitantly with a rapid cell doubling time. Therefore, the proposed *U*_*w*_ provides a better understanding of the water relationships of microorganisms in food preservation.

## Introduction

Microbial response is a critical factor to consider when evaluating the quality and processability of food preservation, as it has significant implications for public health^1,2^. For instance, microbial growth may lead to alteration of the physicochemical properties of foods, which in turn may affect the shelf-life or even become life-threatening in a certain case^3,4^. In general, food is a complex and dynamically heterogeneous system consisting of a myriad of biomaterials and solvents, which can collectively determine the microbial response in final products, i.e., the survival and growth demanded carbon and nitrogen sources are satisfied by sugars and proteins^5^. Water, as a major solvent in foods, can provide a hospitable environment to support microbial growth, whereas removing water by simultaneously transferring heat and mass effectively inhibits the growth of spoilage and pathogenic microorganisms in foods^6,7^. However, such dehydration process seems not a reliable method for food preservation as pathogenic microorganisms have been shown to survive for several days, weeks, and even months or years in low-moisture foods. For example, *Aspergillus flavus* has been detected in baked foods^8^; *Salmonella* and *Cronobacter sakazakii* can contaminate powdered milk^9^. As more cases related to low-moisture foods as a vehicle for the foodborne disease are recorded worldwide, the rationale of microbial growth in food solids becomes a great challenge and needs to be understood, comprehensively.

For half a century, water activity (*a*_*w*_) has become a critical indicator responsible for governing spoilage and pathogenic microorganisms in foods, principally because the measured *a*_*w*_ values generally correlate well with the potential for the growth and metabolic activity of microbes^7^. However, the physicochemical properties of solids in low-moisture food are often time-dependent, and the use of *a*_*w*_ should always consider the possible influences of nonequilibrium situations, such as glassy or crystallizable components, and thus, the *a*_*w*_ alone may be inappropriate to describe the attributes of these systems^10,11^. In low-moisture food matrices, for instance, the glass transition plays an important role in determining the stability and shelf-life of these products. When a food matrix is in its glassy state, it is essentially frozen in time, with very little molecular mobility. This can help to preserve the quality and freshness of the food by slowing down chemical reactions and microbial growth. However, if the food matrix is exposed to moisture and begins to absorb water, it can transition back to its rubbery state and become more susceptible to degradation^12^. Previous studies have reported a temperature range of glass transition (*T*_*g*_) considered as a physicochemical boundary to control the physicochemical properties as well as microbial growth in low-moisture foods as the viscosity of the system increases as *T*_*g*_ increases^13^. Unfortunately, reality does not always appear to follow this scheme. Some xerophytic microbes, e.g., *Aspergillus flavus, Xeromyces bisporus*, *Chrysosporium fastidium* have been found in solid foods and spoilage may not be prevented by keeping the product below its *T*_*g*_ value with high viscous situations^8,14^. Although the *a*_*w*_ is a solvent property and *T*_*g*_ refers to a property related to the structure of the solute matrix, both are strongly dependent on water involved in solid systems. Therefore, knowledge of both *a*_*w*_ and *T*_*g*_ needs a better understanding of the water behavior in solid foods.

The increasing evidence proposed that complementing *a*_*w*_ with water mobility dynamics may be an attribute that deserves further attention as the water mobility in low-moisture foods was highly correlated to many necessary diffusion-limiting processes to the growth and metabolic activity of microorganisms. In the context of water mobility, the NMR can be used to measure the translational diffusion coefficient of water molecules, which is related to the structural relaxation time of the system. The NMR probing of water may be detected by three magnetic nuclei (^1^H, ^2^H, and ^17^O), however, chemical exchanges can occur between ^1^H and ^2^H nuclei as well as cross-relaxation processes with non-water protons which compromises the water mobility measurement. On the other hand, the ^17^O-NMR does not have such complexities. For example, the ^17^O-NMR relaxation studies indicated that the water translational mobility-derived relaxation times (*τ*) were linear with the lag phase of *Staphylococcus aureus*^15^, and the translational mobility of water could provide alternative measures than *a*_*w*_ for predicting the germination of *Aspergillus niger*^16^. Such experimental facts indicate that water mobility is necessary for the transportation of nutrients and metabolites for the growth of microorganisms, and thus, a more dependable and precise quantification of water mobility in low-moisture food needs to be carefully address or propose^13^. Numerous publications have independently used translational relaxation times as a measure of water mobility and the *T*_*g*_ as an indicator of the overall mobility of the food system^17,18^. However, few studies provide descriptive on relationships between water and solids mobility and the *T*_*g*_ in solid foods and their potential applications to effectively inhibit or retard microbial growth.

To take into account the rate of variation in molecular mobility while crossing *T*_*g*_, our previous works have defined the classification for solid-food systems: *Strength* parameter, *S*^19,20^. The *S* concept, identifying an allowable temperature range increases above *T*_*g*_, is a parameter descriptive of the physical state of all components (including water) involved in solid foods as it intuitively expresses the spatiotemporal responsibility of molecules to change in motion. The spatiotemporal responsibility describes the motion state of small sugar molecules or their groups in different phases, which directly related to the dynamic dependence of molecular flow induced by translation of small sugar molecules near *T*_*g*_ when facing temperature changes^21,22^. Besides, the *Deborah* number, which defined as the ratio of the characteristic time scale of a material’s flow to the relaxation time of the material, was applied to provide a useful translation of measured *τ* to real experimental timescales in *S* parameter. It should note that the types and speeds of mobility of water are dependent on temperature and external pressure, and in a solid food, on composition and system kinetic, i.e., changes over temperature and time^20^. Previous studies found a compositional dependence of *S* values in solid foods, which refers to the overall mobility of the system that can be derived from the mobility of individual components^19^. Therefore, such compositional dependence of the *S* value would give a possible approach for revealing the fundamental role that water plays in solid foods. However, the quantification of water mobility in solid foods still lacking in study as the fundamental aspects of compositional dependence of *S* still needs to be discussed and extended by considering the application of classical thermodynamics.

Glucose, a commonly used food ingredient, often presents in glass formers and can determine the thermodynamic properties and affect the water mobility in sugar/protein solids owing to its hydrophilic and nonequilibrium nature^23^. Whey protein isolates (WPI), which can effectively prevent the phase and state transition of glassy compounds, is widely added into food as a commercial stabilizer in the industry^24^. In this study, the glucose and WPI were chosen to composite solid-food model after lyophilizing as yeast can grows rapidly when glucose and protein are present, and the *Debaryomyces hansenii* (*D. hansenii*) was chosen as a target yeast and inoculated in the glucose/WPI solid matrices for incubating 36 h at *a*_*w*_ ≥ 0.76 and 30 °C. The water sorption and thermodynamic properties of amorphous glucose/WPI solid matrices were measured after equilibrium at various *a*_*w*_ and 30 °C, while the thermodynamic discussion was complemented in explicating the composition dependence of *S*. The mobility difference between system-involved water and liquid pure water was calculated and gave a definition of an innovative indicator: water usability, *U*_*w*_. The potential usage of water usability in modulating microbial growth was also investigated by relating *U*_*w*_ values to the growth characteristics of *D. hansenii*. In this paper, the proposed *U*_*w*_, which contributed to a combinative parameter of thermodynamic properties and water mobility dynamics, can better understand the water relationships of microorganisms in low-moisture food preservation.

## Results and discussion

### Water sorption isotherms

**Fig. 1:**
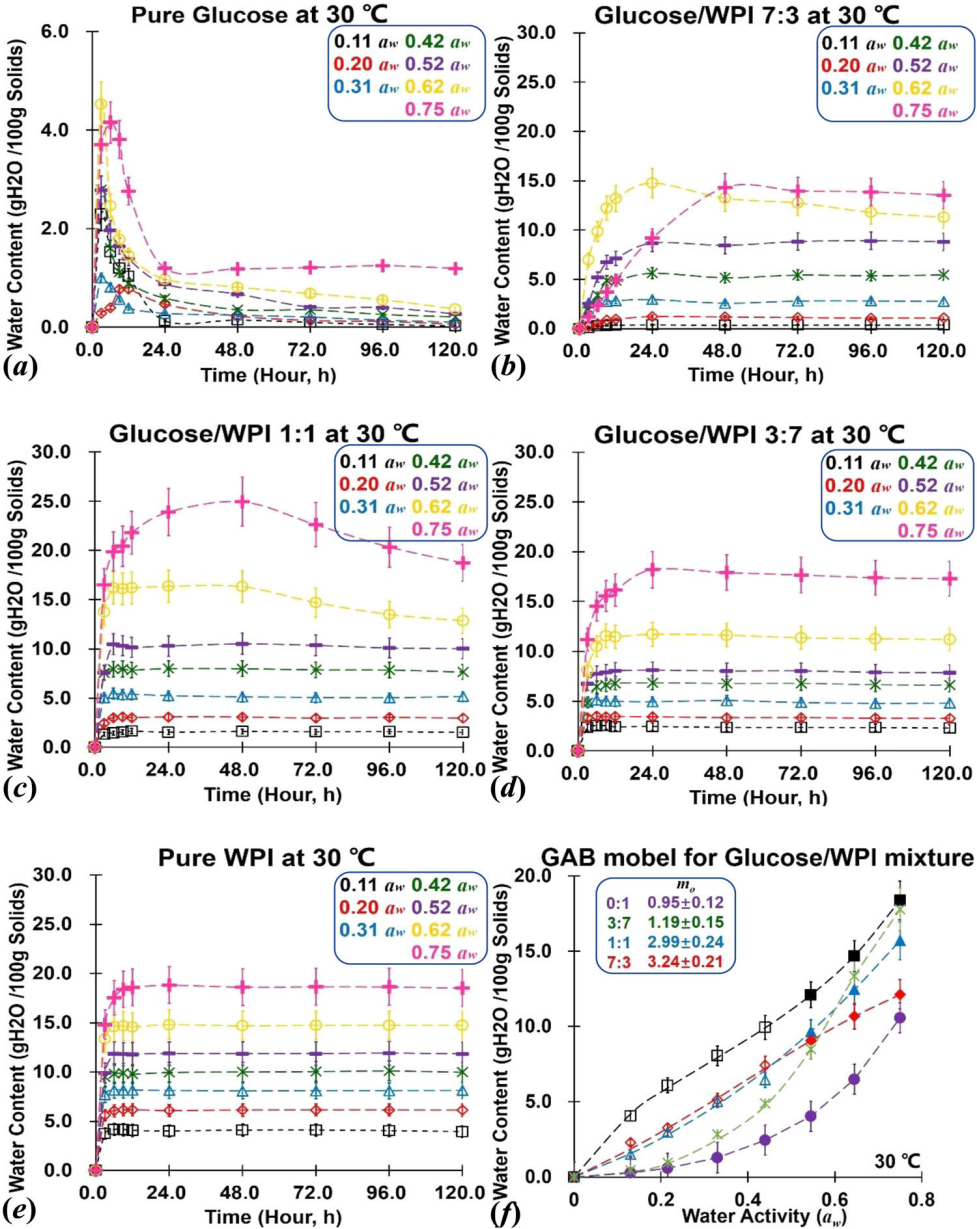
The water sorption isotherms for glucose/WPI solid. The water sorption isotherms for glucose/WPI solid matrices with mass ratios of 1:0, 7:3, 1:1, 3:7, and 0:1 at *a*_*w*_ from 0.11 to 0.75 under 30 °C (**a**–**e**). The GAB model and monolayer sorption value (*m*_0_) for each sample were given in (**f**), where the empty and solid symbols represent the experiment and calculated sorption data on the basis of Eq. (1), respectively. The error bar on the bar charts represents the standard error.

The steady-state water sorption data and corresponding sorption isotherms for prepared glucose/WPI solid matrices (1:0, 7:3, 1:1, 3:7, and 0:1; w/w) after equilibrium at various storage conditions (0.11–0.76 *a*_*w*_ at 30 °C) were shown Fig. 1 and given in Supplementary Table 1. The water sorption data of each sample was slightly lower than previous reports, probably owing to the variation in the dehydration process, sorption time, and storage temperature^25^. In this study, the crystallization of amorphous glucose was observed as sorption data rapidly decreased after 12 h of storage at all studied *a*_*w*_ and 30 °C (Fig. 1a). This was caused by the non-crystallized portion rejected sorbed water molecules from glucose crystals formation, which induced desorption behavior^21^. No water release was found in glucose/WPI matrices with studied mass ratios at *a*_*w*_ below 0.44 and 30 °C, whereas the lost of sorbed water was observed and slightly became rapid in glucose/WPI with 7:3 and 1:1 (w/w) at *a*_*w*_ above 0.44 (Fig. 1b–d). This result was induced by the WPI-derived physical-blocking effects reduced molecular diffusion, then, induced the partial crystallization of amorphous glucose. However, the high *a*_*w*_ condition might weaken crystallization owing to a water mobility-driven plasticization^26^. Moreover, crystallization was merely observed in pure WPI over the whole studied *a*_*w*_ range at 30 °C during 120 h of storage (Fig. 1e). In this study, the water sorption data in glucose/WPI solid matrices exhibited a result of fractional quantities at *a*_*w*_ ≤ 0.44 and 30 °C, while the GAB-derived monolayer sorption (*m*_0_) value of glucose/WPI solid matrices showed composition-dependent characteristic (Fig. 1f). This indicated that the phase separation occurred in glucose/WPI matrices during water sorption testing, where water was individually hydrogen bonding to protein and glucose; thus, fewer hydrogen bonds might exist between protein and lactose in mixtures^27^. Potes and others^28^ have reported a water additive principle in sugar/protein solid matrices, which refers to the steady-state water content of non-crystalline sugar that could be calculated through pure protein and high protein-containing systems based on phase separation and GAB sorption isotherms (Eq. 1). In Eq. (1), *W*_*t*_ is the total equilibrium water content in the system; *n*_1_, ⋯, *n*_*n*_ is the mass fraction of each component in the system; *W*_1_, ⋯, *W*_*n*_ is the water contents sorbed by each component. Therefore, the steady-state water contents for non-crystalline glucose from 0.11 to 0.75 *a*_*w*_ and glucose/WPI solid matrices (7:3 and 1:1, w/w) from 0.52 to 0.75 *a*_*w*_ were calculated and given in Supplementary Table 1. Since glucose is a readily crystallizable biomaterial due to its low *T*_*g*_ and high solubility nature, the above finding and calculated sorption data for non-crystalline glucose in sugar/protein solid matrices have great importance in the development of processing and shelf-life control procedures for glucose-containing solid foods.

1 $${W}_{t}={n}_{1}{W}_{1}+,\cdots ,+{n}_{n}{W}_{n}$$

### Thermodynamic properties

**Fig. 2:**
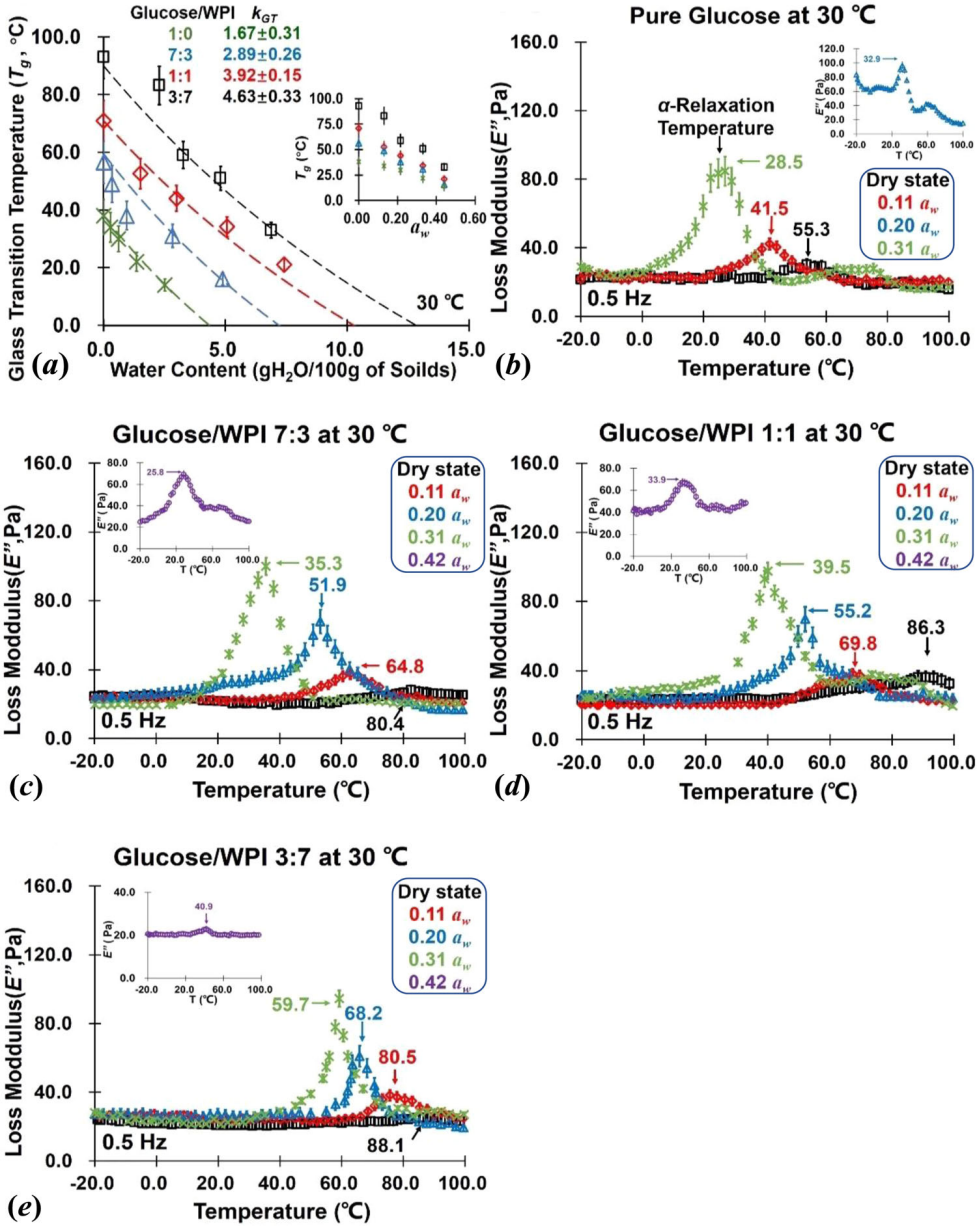
The thermal and dynamic properties for glucose/WPI solid matrices. The calorimetric onset-*T*_*g*_ (**a**) and the DMA spectra of *E*″ at 0.5 Hz (**b**–**e**) of glucose/WPI solid matrices (1:0, 7:3, 1:1, 3:7, and 0:1, w/w) stored from dry state to 0.44 *a*_*w*_ at 30 °C. The *T*_*g*_ values against corresponding water sorption data were fitted by *GT* equation, and the *T*_*α*_ values were characterized from the peak temperature of *E*″^28^. The error bar on the bar charts represents the standard error.

The calorimetric onset-*T*_*g*_ values of glucose/WPI solid matrices (1:0, 7:3, 1:1, 3:7, and 0:1; w/w) after storage at *a*_*w*_ ≤ 0.44 and 30 °C determined by DSC were given in Table 1. In this paper, the *T*_*g*_ values of amorphous glucose were agreed with Simperler and others^29^, and a slightly difference may be found in sample preparation, environmental factors, or measurement methods. At the high *a*_*w*_ range (≥0.54 *a*_*w*_), it was not easy to measure the *T*_*g*_ value of each sample due to the crystallization occurring, and *T*_*g*_ was not observed in pure WPI because the vitrification transformation of protein was not obvious. It should note that the glass transition occurred only in non-fatty solid components and was affected by water-mobility-induced plasticization^26^. In Table 1, hence, the *T*_*g*_ value of the systems with higher WPI content was higher than pure glucose at studied conditions, which because of the stronger water binding ability of WPI may weak the mobility of water. The relationship between *T*_*g*_ values and water content (Dry ~ 0.44 *a*_*w*_) of glucose/WPI solid matrices (1:0, 7:3, 1:1, and 3:7, w/w) was correlated by *GT* model (Eq. 8) and shown in Fig. 2a. The *T*_*g*_ data of non-crystalline samples were calculated by applying the extrapolating water sorption data from GAB isotherms into *GT* model. It should note that the *k*_*GT*_ in *GT* model was related to the strength of interaction between components in the mixture, namely hydrogen bond and charge transfer interaction, which could be calculated by the mass fraction of sorbed water in the glucose/WPI solid matrices^21^. In Fig. 2a, similarly, the *k*_*GT*_ value increased with the increase of WPI content in the system. This result indicated that the mobility of water molecules was captured or trapped by the presence of protein via water-bonding sites, and WPI could disturb the mobility of water in glucose/WPI solid matrices in agreement with water sorption results.

**Table 1:** The calorimetric onset-*T*_*g*_, *T*_*α*_, WLF-constant *C*_1_ and *C*_2_, and *S* value for glucose/WPI solid matrices (1:0, 7:3, 1:1, 3:7, and 0:1; w/w) after equilibrium at studied *a*_*w*_ and 30 °C.

		*a*_*w*_	*T*_*g*_ (°C)	*T*_*α*_ (°C)	*C*_1_	*C*_2_	*S* (°C)
Glucose	Pure	Dry	38.2 ± 1.1*	54.2 ± 2.8^a^**	−0.3 ± 1.7^a^	−18.1 ± 3.6^a^	16.9 ± 1.6^a^
		0.11 ± 0.01	34.6 ± 3.2^a^	44.1 ± 3.1^b^	−3.6 ± 5.3^b^	−16.2 ± 4.3^b^	15.0 ± 4.7^b^
		0.20 ± 0.03	30.8 ± 4.4^b^	33.7 ± 2.5^c^	6.5 ± 2.8^c^	7.9 ± 3.7^c^	12.6 ± 2.2^c^
		0.31 ± 0.01	25.5 ± 2.8^c^	28.2 ± 5.1^d^	−0.9 ± 3.7^d^	−10.8 ± 4.5^d^	8.6 ± 1.3^d^
		0.42 ± 0.02	12.4 ± 4.2^d^	–	–	–	6.7 ± 2.4***
		0.52 ± 0.050		–	–	–	5.1 ± 4.5
		0.62 ± 0.01		–	–	–	4.6 ± 3.1
		0.75 ± 0.02		–	–	–	3.0 ± 1.7
		0.81 ± 0.03		–	–	–	2.1 ± 1.2
		0.92 ± 0.04		–	–	–	1.6 ± 1.5
		1.00 ± 0.08		–	–	–	1.5 ± 1.2
Glucose/WPI	7:3	Dry	56.5 ± 2.4^a^	80.3 ± 4.7^a^	−5.9 ± 1.6^a^	−81.5±3.7^a^	31.3 ± 0.8^a^
		0.11 ± 0.01	45.4 ± 3.4^b^	67.6 ± 5.3^b^	−1.5 ± 2.5^b^	−25.9±4.7^b^	25.2 ± 1.2^b^
		0.20 ± 0.03	33.2 ± 3.1^c^	66.8 ± 3.5^c^	5.6 ± 3.4^c^	18.9±3.1^c^	19.4 ± 2.5^c^
		0.31 ± 0.01	24.9 ± 2.3^d^	30.2 ± 2.4^d^	8.3 ± 4.7^d^	16.2±1.9^d^	11.5 ± 0.9^d^
		0.42 ± 0.02	16.5 ± 3.6^e^	26.7 ± 4.2^e^	−1.8 ± 3.7^e^	−8.5±1.2^e^	7.5 ± 1.4^e^
		0.52 ± 0.050		–	–	–	5.6 ± 3.3
		0.62 ± 0.01		–	–	–	4.1 ± 2.5
		0.75 ± 0.02		–	–	–	3.4 ± 2.2
		0.81 ± 0.03		–	–	–	2.5 ± 0.9
		0.92 ± 0.04		–	–	–	1.8 ± 1.3
		1.00 ± 0.08		–	–	–	1.6 ± 2.1
Glucose/WPI	1:1	Dry	70.9 ± 2.2^a^	85.4 ± 3.1^a^	−6.5 ± 4.7^a^	−36.7 ± 2.9^a^	38.7 ± 2.5^a^
		0.11 ± 0.01	52.6 ± 1.5^b^	62.4 ± 4.6^b^	−4.5 ± 3.8^b^	−29.9 ± 4.3^b^	29.2 ± 0.8^b^
		0.20 ± 0.03	44.0 ± 2.8^c^	45.5 ± 5.8^c^	−6.8 ± 5.1^c^	−35.6 ± 6.4^c^	20.4 ± 1.0^c^
		0.31 ± 0.01	34.2 ± 3.0^d^	42.3 ± 3.7^d^	9.6 ± 4.7^d^	25.6 ± 2.3^d^	17.5 ± 2.6^d^
		0.42 ± 0.02	22.1 ± 4.3^e^	32.9 ± 4.5^e^	−3.8 ± 1.1^e^	−13.7 ± 2.8^e^	12.9 ± 3.1^e^
		0.52 ± 0.050		–	–	–	11.1 ± 3.2
		0.62 ± 0.01		–	–	–	9.3 ± 0.5
		0.75 ± 0.02		–	–	–	7.6 ± 1.5
		0.81 ± 0.03		–	–	–	6.4 ± 2.1
		0.92 ± 0.04		–	–	–	5.1 ± 3.8
		1.00 ± 0.08		–	–	–	5.3 ± 2.5
Glucose/WPI	3:7	Dry	93.2 ± 1.2^a^	88.1 ± 4.5^a^*	−0.1 ± 0.8^a^	−41.1 ± 0.6^a^	41.7 ± 1.5^a^
		0.11 ± 0.01	83.1 ± 2.1^b^	80.1 ± 2.8^b^	−9.6 ±1.4^b^	−38.1 ± 5.7^b^	37.6 ± 2.2^b^
		0.20 ± 0.03	59.1 ± 4.1^c^	68.2 ± 1.9^c^	−7.4 ± 2.8^c^	−29.4 ± 2.9^c^	27.9 ± 1.9^c^
		0.31 ± 0.01	50.1 ± 2.0^d^	59.7 ± 4.4^d^	−6.7 ± 2.7^d^	−62.4 ± 5.2^d^	23.4 ± 3.1^d^
		0.42 ± 0.02	33.8 ± 3.0^e^	40.9 ± 5.3^e^	−5.5 ± 1.8^e^	−21.6 ± 7.2^e^	20.5 ± 0.9^e^
		0.52 ± 0.050		–	–	–	18.3 ± 1.2
		0.62 ± 0.01		–	–	–	16.7 ± 2.5
		0.75 ± 0.02		–	–	–	15.0 ± 3.0
		0.81 ± 0.03		–	–	–	14.4 ± 0.5
		0.92 ± 0.04		–	–	–	13.8 ± 1.1
		1.00 ± 0.08		–	–	–	6.5 ± 1.1

*Values are means ± SDs (*n* = 3).

**Significant analysis at two-sided t-test, *p* < 05; superscript letters a–f refer to the statistical significance.

***The prediction *S* value.

Figure 2b–e showed the DMA spectrum (*E*″ peak frequency is 0.5 Hz) of studied glucose/WPI solid matrices (1:0, 7:3, 1:1, 3:7, and 0:1; w/w) after storage at *a*_*w*_ ≤ 0.44 and 30 °C determined by DMA. In the experimental temperature range, the *E*″ peak of glucose decreased and widened with the decrease of *a*_*w*_, which was caused by the change of dynamic-mechanical properties of amorphous materials affected by water, and the changes in water content could change the molecular mobility of solid matrices, and thus, interfering with the peak of *E*″ in multi-frequency testing mode. At *a*_*w*_ ≤ 0.44 and 30 °C, the *E*″ peak intensity of glucose/WPI matrices decreased with the increase of WPI content. The above result suggests that water mobility-induced plasticization in studied solid matrices could be disturbed by the presence of proteins, as noted above. The peak temperature of *E*″ was used to determine the *T*_*α*_ of the mixture (Table 1). The *T*_*α*_ referred to the temperature point at which the mobility of amorphous sugar molecules was inphase with a certain frequency^28^. In this study, the *T*_*α*_ of glucose/WPI solid matrices decreased with the increase of *a*_*w*_, while the increase of WPI content could increase the *T*_*α*_ value of the system (Table 1). The physical state of the mixture system was strongly affected by the water content, which changed the molecular mobility of glucose and disrupted *T*_*α*_. According to DMA results, therefore, the mobility of water could affect the molecular interactions, *α-*relaxation, and *T*_*α*_ of solid foods.

### Molecular mobility measurement

**Fig. 3:**
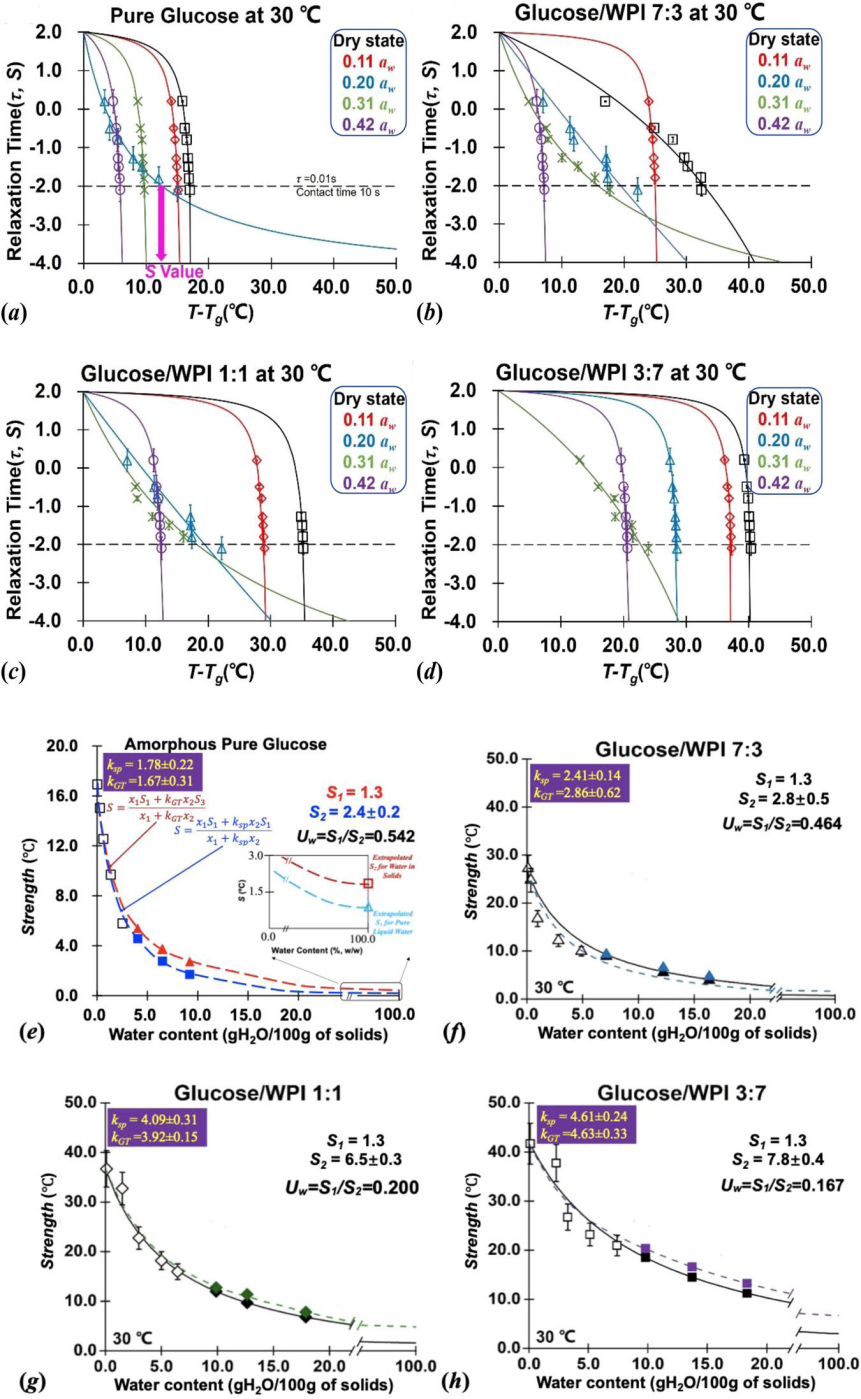
Strength (*S*) value and water usability (*U*_*w*_) for glucose/WPI solid matrices. Strength (*S*) value for glucose/WPI solid matrices (1:0, 7:3, 1:1, and 3:7, w/w) under various *a*_*w*_ (dry to 0.44 *a*_*w*_) and 30 °C (**a**–**d**). The relationship between *S* and corresponding water content for studied solid matrices was fitted by Eq. (11) on the basis of *k*_*sp*_ (solid line) and *k*_*GT*_ (dash line) shown in (**e**–**h**). The *S* value of water involved in studied solid matrices (*S*_1_) was extrapolated and compared to the literature data of liquid pure water (*S*_2_) to interpret the water usability^37^. The error bar on the bar charts represents the standard error.

In Fig. 3a–d, the relationship between DMA-derived α-relaxation time (*τ*) and temperature difference (*T*_*g*_ − *T*_*α*_) for studied glucose/WPI solid matrices (1:0, 7:3, 1:1, and 3:7; w/w) that equilibrium at low *a*_*w*_ (dry ~ 0.44 *a*_*w*_) and 30 °C was successfully fitted by William–Landel–Ferry (WLF) equation (Eq. 9) with materials-specific constants *C*_1_ and *C*_2_. It should note that the WLF constants *C*_1_ and *C*_2_ often involve different physical meanings for describing solids flow characteristics, where *C*_1_ is a time factor that refers to the maximum number of log decades for the change in *τ* as anchored to *T*_*g*_ and *C*_2_ expresses a theoretical temperature (°C) for infinite *τ*^21^. Most of the WLF relationships for studied solid matrices showed down concavity as their *C*_1_ and *C*_2_ values were numerical negative, which both increased concomitantly with *a*_*w*_ increases (Table 1). This resulted from the water mobility-induced plasticization that could decrease the *T*_*g*_ of solid systems, where *τ* rapidly dropped in several logarithmic decades when the temperature was above *T*_*g*_. It should note that the determination of *τ* in relaxation data measured at different frequencies by DMA can refer to the time factor corresponding to the material response to a change in internal or external thermodynamic conditions such as temperature and *a*_*w*_, which can provide a new approach for the description of the mobility dynamics for food constituents^13^. Previous studies reported a WLF constant derived *S* concept was a measurement of structural transformation and mobility resistance, which could be used to determine the molecular mobility of solid foods^20^. In this study, the *S* values of the glucose/WPI solid matrices (1:0, 7:3, 1:1, and 3:7; w/w) increased with the increase of WPI content but decreased with *a*_*w*_ increases (Table 1). This indicated that water mobility-induced plasticization could reduce the *T*_*g*_ and increase the apparent molecular mobility of the system, whereas the presence of WPI delayed the apparent molecular mobility owing to its physical-blocking effects and water-bonding sites reduced the mobility of sugar and water molecules. Previous studies have proposed a practical equation (Eq. 11) to describe the relationship between water content and *S* in amorphous sugar/protein systems^19^. Such relationship was also applied for describing the influence on numerical *S* values and water content of glucose/WPI solid matrices and achieved an excellent fitting performance in this paper (Fig. 3e–h). The present work confirms that the *Strength* approach is universal and can apply to hygroscopic monosaccharides/protein systems, which possess a practical potential in the fabrication of dampproof foods as well as improving the processability, quality, and shelf-life of solid foods.

### Mobility dynamics of water

The constant *k*_*sp*_ in Eq. (11) implies the extent of water mobility-induced plasticization on amorphous food systems, in which a small *k*_*sp*_ refers to a less strong structure^21^. In this study, similarly, the constant *k*_*sp*_ of studied glucose/WPI solid matrices were increased by the presence of WPI owing to the extent of plasticization was weakened by protein. Despite the compositional dependence of *S* values in Eq. (11), the *k*_*sp*_ of studied samples were very similar but only slightly higher than the *GT* constant *k*_*GT*_, numerically (Figs. 2a and 3e–h). Although *k*_*sp*_ and *k*_*GT*_ were respectively calculated on the basis of the *T*_*g*_ and mobility dynamics, their numerical similarity is not entirely coincidental. The thermodynamic discussion on the effects of composition on the *S* value in Eq. (11) and corresponding constant *k*_*sp*_ was carefully explicated below. For simplicity, the pure amorphous glucose was taken as an example, where the respective mole fractions of the two components (glucose and water) in the system are denoted as *x*_1_ and *x*_2_, *C*_*p*1_ and *C*_*p*2_ denote the molar heat capacities, and the molar entropies of these pure components are designated in turn as $\bar{S}$_1_ and $\bar{S}$_2_. The molar entropy ($\bar{S}$_*mix*_) of the binary system can be written generally as ${\bar{S}}_{{mix}}={x}_{1}{\bar{S}}_{1}+{x}_{2}{\bar{S}}_{2}+\triangle {\bar{S}}_{{mix}}$. To be specific, the $\triangle {\bar{S}}_{{mix}}$ includes excess entropy (solely conformational for simplest case) changes associated with mixing the two components. Couchman and Karasz^30^ have treated glass transition as an Ehrenfest second-order transition on the basis of the configurational entropy theory and used the thermodynamic characteristic continuity and discontinuity conditions, together with some simple explicit assumptions and approximations, to provide relations expressing the *T*_*g*_ of the binary mixture in terms of the *T*_*g*1_ and *T*_*g*2_ of the individual pure components. Based on the above assumption, the composition of the system is fixed it then follows that $\triangle {\bar{S}}_{{mix}}$ is continuous at *T*_*g*_ range, which pointed to a circumstance where the character and extent of specific interactions barely changed at *T*_*g*_ as non-conformational contributions to the excess entropy of mixing. Therefore, the $\bar{S}$_*mix*_ of the binary system can be written generally in Eq. (2). As noted above, the *S* parameter refers to an allowable temperature increase above the calorimetric onset-*T*_*g*_ for non-crystalline materials. Meanwhile, Slade and others^11^. have reported that the first-order transition for non-crystalline sugars, such as crystallization, was ~50 °C above the calorimetric *T*_*g*_ value. In this study, we found that the *S* value for pure anhydrous glucose was 16.9 °C above its calorimetric onset-*T*_*g*_, while the presence of water decreased the *S* value of amorphous glucose (Table 1). This result proved that the *S* should locate in the Ehrenfest second-order transition range, which indicated the assumption suggested by Couchman and Karasz^30^. can still hold to explain the compositional dependence of the *S* parameter. Therefore, let *T*, *T*_1_, and *T*_2_ denote temperature points above the calorimetric onset-*T*_*g*_ for the system and pure individual components within respectively glass transition range (*T*_*g*_, *T*_*g*1_, and *T*_*g*2_). The part of total molar entropy (Eq. 2), excluding excess entropy could be written generally as Eq. (3), where *S*_*p*_, *S*_1_, and *S*_2_ refer to the *S* parameters for the binary system and involved components.

2 $${\bar{S}}_{{mix}}={x}_{1}\left[{\bar{S}}_{1}+{\int }_{{T}_{g1}}^{{T}_{g}}\frac{{C}_{p1}}{T}{dT}\right]+{x}_{2}\left[{\bar{S}}_{2}+{\int }_{{T}_{g2}}^{{T}_{g}}\frac{{C}_{p2}}{T}{dT}\right]$$

3 $${\bar{S}}_{{mix}}={x}_{1}\left[{\bar{S}}_{1}+{\int }_{{T}_{1-}{T}_{g1}}^{T-{T}_{g}}\frac{{C}_{p1}}{T}{dT}\right]+{x}_{2}\left[{\bar{S}}_{2}+{\int }_{{T}_{2-}{T}_{g2}}^{T-{T}_{g}}\frac{{C}_{p2}}{T}{dT}\right]={x}_{1}\left[{\bar{S}}_{1}+{\int }_{{S}_{1}}^{{S}_{p}}\frac{{C}_{p1}}{T}{dT}\right]+{x}_{2}\left[{\bar{S}}_{2}+{\int }_{{S}_{2}}^{{S}_{p}}\frac{{C}_{p2}}{T}{dT}\right]$$

Equation (3) can be obtained for both the glassy and rubbery states, where the *C*_*p*_ undergoes a finite discontinuity at the transition^31^. Since ${\bar{S}}_{1}$ and ${\bar{S}}_{2}$ are continuous at respectively *T*_*g*1_ and *T*_*g*2_, the continuity of ${\bar{S}}_{{mix}}$ at *T*_*g*_, and the approximation that the transition isobaric heat capacity increments $\triangle {C}_{p1}$ and $\triangle {C}_{p1}$ are temperature independent provides the expression in Eq. (4). If the further approximation ln(1 + *x*) ≈ *x* is valid, the Eq. (4) was rearranged as Eq. (5), where the meaning of *k*_*sp*_ can also be written as the changes of heat capacities of individual components for the binary system. Consequently, the above classic thermodynamic discussion explicated that the *k*_*sp*_ and *k*_*GT*_ were not only similar in numerical value but shared the same thermodynamic meanings.

4 $${\mathrm{ln}S}_{p}=\frac{{x}_{1}\mathrm{ln}{S}_{1}+\frac{\triangle {C}_{p2}}{\triangle {C}_{p1}}{x}_{2}\mathrm{ln}{S}_{2}}{{x}_{1}+\frac{\triangle {C}_{p2}}{\triangle {C}_{p1}}{x}_{2}}$$

5 $${S}_{p}=\frac{{x}_{1}{S}_{1}+\frac{\triangle {C}_{p2}}{\triangle {C}_{p1}}{x}_{2}{S}_{2}}{{x}_{1}+\frac{\triangle {C}_{p2}}{\triangle {C}_{p1}}{x}_{2}}$$

### Water usability, *U*_*w*_

Although the thermodynamic discussion explicitly explains the thermodynamic meaning of *k*_*sp*_ and *k*_*GT*_, their slight numerical difference is still unignorable and needs to be clarified fully. In Fig. 3e–h, the extrapolation line for glucose/WPI solid matrices (1:0, 7:3, 1:1, and 3:7, w/w) was fitted by Eq. (11) using *k*_*GT*_ to replace *k*_*sp*_ as constant. In Fig. 3e–h, we noticed that the extrapolated *S* value by *k*_*GT*_ for water involved in solid matrices was higher than the extrapolated *S* value calculated by *k*_*sp*_, which chose the literature *S* value from liquid pure water^19^. It should be noted that the WLF equation and NMR spectroscopy are complementary techniques, which can provide valuable information on the dynamics of water. However, the comparison of the ^17^O-NMR technique with WLF equation is valid for translational mobility, as ^17^O-NMR is less affected by chemical exchange and cross-relaxation processes than ^1^H and ^2^H NMR. Schmidt^17^ has pointed out that the translational mobility of water decreases when a component is added, and the magnitude of the decrease depends on the number, amount, and nature of the solute and processing methods based on NMR studies. Similarly, the thermodynamic discussion indicated that the water in solid matrices may exhibit different translational mobility dynamics than those of liquid pure water as the states of water in systems vary due to the hydrogen bonding, capillary, crystallized, etc. The varying mobility dynamic of water might contribute to fabricating the food structures and many other functional abilities for the whole systems, such as nutrients transportation, microbial growth, structural fabrications, and so on^32^. Therefore, the *k*_*GT*_ extrapolated *S* value could give a better description of the mobility dynamics of water involved in systems than the literature *S* value of liquid pure water. As noted above, the quantification of water mobility in solid foods requires considering both thermodynamic properties and water mobility dynamics. On the basis of the molecular mobility difference between water involved in solid matrices and liquid pure water in their *T*_*g*_ range, we introduced water usability (*U*_*w*_) to give a measure for the mobility of water involved in solid matrices (Eq. 6). In Eq. (6), the *S*_1_ and *S*_2_ refers to the molecular mobility of liquid pure water and water involved in solids. Like *a*_*w*_ concept, it should be noted that the *U*_*w*_ value are between 0 to 1, where the more of free mobility water in the systems the higher of the *U*_*w*_ values. Compared to *a*_*w*_ and *T*_*g*_, the proposed *U*_*w*_ could give a better interpretation of the changes of water mobility in solid foods.

6 $${U}_{w}=\frac{{S}_{1}}{{S}_{2}}$$

### Microbial growth and *U*_*w*_

**Fig. 4:**
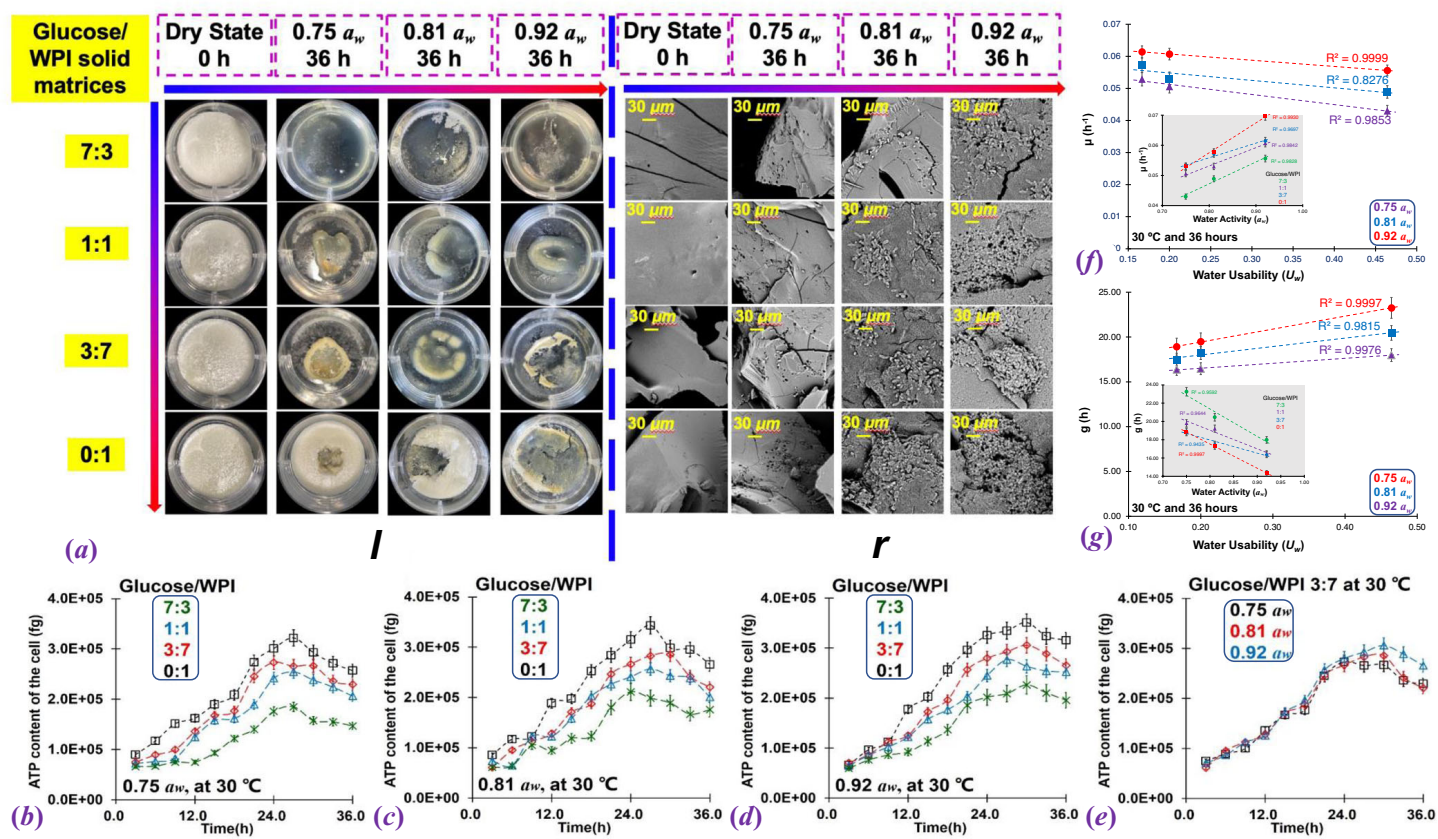
The growth of *D. hansenii* in glucose/WPI solid matrices and its relationship with *U*_*w*_. Photos (left, l) and SEM images (right, r) for the growth of *D. hansenii* in glucose/WPI solid matrices (7:3, 1:1, 3:7, and 0:1, w/w) after 36 h of incubation at 0.75 to 0.92 *a*_*w*_ and 30 °C (**a**). The corresponding growth curves of *D. hansenii* were shown from (**b**) to (**e**), and the relationship between the *U*_*w*_*, a*_*w*_, specific growth rate (*μ*), and cell doubling time (*g*) of *D. hansenii* were shown from (**f**) to (**g**). The error bar on the bar charts represents the standard error.

The appearance of *D. hansenii* inoculated glucose/WPI solid matrices (7:3, 1:1, 3:7, and 1:0, w/w) after 36 h incubating at *a*_*w*_ varying from 0.75 to 0.92 *a*_*w*_ and 30 °C were shown in Fig. 4a left. It should note that none of *D. hansenii* could survive in pure glucose at all studied high *a*_*w*_ as the extreme osmotic pressure in this study, whereas the pure WPI solids would cultivate the *D. hansenii* as the impurities included in WPI powder might provide the growth factors for microbes. Previous studies reported that the presence of excess water surrounding the sugar/protein composite solids can create a continuous liquid phase allowing fast exchange among different regions of the sample as well as weakening the physical structure^33^. Similarly, the water sorption-induced structural collapse was observed in all studied glucose/WPI solid matrices at mass ratios of 7:3, 1:1, and 3:7 after 36 h of incubating at *a*_*w*_ varying from 0.75 to 0.92 *a*_*w*_ and 30 °C (Fig. 4a left). Besides, such morphological deterioration was also found in pure WPI solids, which disagreed with Fan and Roos^27^. who pointed out that the amorphous protein might exhibit a stronger structure with no collapse phenomenon after equilibrium at a high *a*_*w*_ range. Based on the SEM observation, the colony of *D. hansenii* in glucose/WPI solid matrices (7:3, 1:1, and 3:7, w/w) was found after 36 h of incubation at *a*_*w*_ varying from 0.75 to 0.92 *a*_*w*_ and 30 °C, where the size of the strain increased with the increasing of WPI content (Fig. 4a right). Besides, in this study, the SEM results have shown that *D. hansenii* can grow not only on the surface of the sample, but also inside the freeze-dried sample (Fig. 4a right). This caused by the structure of freeze-dried samples are porous, which can provide the oxygen necessary for *D. hansenii* survival. Therefore, the *D. hansenii* that thrive in WPI solids could use the nutrients and release water through metabolisms, e.g., glycometabolism and cellular respiration, thus, induce the structural collapse in pure WPI solids.

The growth characteristics of *D. hansenii* inoculated in glucose/WPI solid matrices (7:3, 1:1, 3:7, and 0:1, w/w) were monitored in 36 h of incubation at *a*_*w*_ varying from 0.75 to 0.92 and 30 °C by using ATP fluorescence detector (Fig. 4b–d). The growth rate (*μ*) and cell doubling time (*g*) of *D. hansenii* were calculated via Eqs. (12) and (13) and given in Table 2. It should be noted that *D. hansenii* is known to produce glycerol, which can plasticize its growth surroundings without changing *a*_*w*_. However, the experimental conditions designed in this study cannot meet the requirement for yeast to produce enough amount of glycerol to plasticize the solid matrices as *D. hansenii* can only produce glycerol as a compatible solute to help it survive in environments with low *a*_*w*_ (~0.61 *a*_*w*_)^34^. The cell content of *D. hansenii* in glucose/WPI solid matrices increased with the increase of WPI content that was consistency with SEM observation (Fig. 4b–d). This result indicated that the growth of *D. hansenii* was more vigorous as it entered the logarithmic growth phase earlier and rapidly reached the stable phase in a high protein-containing solids. Nevertheless, the tendency of the cell content for *D. hansenii* inoculated in glucose/WPI solids was overlapped at studied *a*_*w*_ ranges (Fig. 4e). It is generally accepted that the tolerance and ability of xerophilic microbes to enable them to survive and grow at reduced *a*_*w*_ rely on a common approach, which indicated that the intracellular accumulation of compatible solutes, e.g., glycerol, arabitol, and mannitol, to balance the *a*_*w*_ inside the cell against the external *a*_*w*_^8^. Therefore, the microbial growth was highly dependent on the nature of the solutes and less dependent on *a*_*w*_. Previous studies have reported that *T*_*g*_ is a physicochemical boundary to control microbial growth in low-moisture foods as the viscosity of the system increases as *T*_*g*_ increases^12,13^. For example, the decrease in solute mobility between glass-forming systems may affect the growth of *S. aureus* as the increasing of *T*_*g*_, which in turn decreases the mobility of nutrients transportation and retards the metabolized activity^13^. However, increasing evidence has pointed out that the high viscosity can enhance the growth of microorganisms in the high-solid system as the slowing of diffusion rate by improving the *T*_*g*_ will making the system more stable and providing better nutrient sources for microorganisms^17^. These experimental findings implied that the growth of microorganisms seems not to correlate with the *T*_*g*_ and its-related viscosity variations of media. Since the water mobility was highly correlated to many important diffusion-limiting processes for microbial growth and metabolic activity in low-moisture foods, in this study, the relationship between the *U*_*w*_, *μ*, and *g* value of *D. hansenii* inoculated in glucose/WPI solid matrices (7:3, 1:1, 3:7, and 0:1; w/w) after incubation at 0.75–0.92 *a*_*w*_ and 30 °C until 36 h were shown in Fig. 4f–g. The *μ* increased with the increase of the *U*_*w*_ value, whereas the *g* value decreased with the increasing of the *U*_*w*_. In Fig. 4f–g, we found that both *U*_*w*_ and *a*_*w*_ are highly correlated with the *μ* and *g* of *D. hansenii*. Nevertheless, the *a*_*w*_ concept has its theoretical shortcomings, such as errors in determining relative saturation vapor pressure, and the inability of *a*_*w*_ to accurately measure it. We believe the *U*_*w*_ has the potential to replace *a*_*w*_ in regulating microbial growth and can be considered as a refinement and supplement to the *a*_*w*_ theory. Therefore, the mobility difference between water involved in foods and liquid pure water can provide a better understanding of the water relationships of microorganisms in low-moisture food preservation.

**Table 2:** The specific growth rate (*μ*) and cell doubling time (*g*) of *D. hansenii* in glucose/WPI solid matrices (7:3, 1:1, 3:7, and 0:1, w/w) after storage at 0.75–0.92 *a*_*w*_ and 30 °C for 36 h.

*a*_*w*_		Glucose/WPI 7:3	Glucose/WPI 1:1	Glucose/WPI 3:7	Glucose/WPI 0:1
0.75 ± 0.02	*μ* (h^−1^)	0.0430 ± 0.0423^a^*	0.0506 ± 0.0548^a^	0.0529 ± 0.0045^a^	0.0531 ± 0.0521^a^
	*g* (h)	23.2548 ± 0.2462A**	19.8073 ± 0.0231^A^	18.9199 ± 0.1213^A^	18.8173 ± 0.0251^A^
0.81 ± 0.03	*μ* (h^−1^)	0.0489 ± 0.0655^b^	0.0530 ± 0.0137^b^	0.0572 ± 0.0213^b^	0.0577 ± 0.0125^b^
	*g* (h)	20.4584 ± 0.3231^B^	19.2197 ± 0.0152^B^	17.4873 ± 0.4463^B^	17.3128 ± 0.2657^B^
0.92 ± 0.04	*μ* (h^−1^)	0.0556 ± 0.0264^c^	0.0607 ± 0.0765^c^	0.0614 ± 0.0321^c^	0.0697 ± 0.0216^c^
	*g* (h)	17.9829 ± 0.4655^C^	16.4681 ± 0.4355^C^	16.3722 ± 0.5418^C^	14.3465 ± 0.2651^C^

*Significant analysis at two-sided t-test, *p* < 05; superscript letters a–c and A–C refer to the statistical significance.

**Values are means ± SDs (*n* = 3).

In this study, consequently, the water sorption isotherms, thermodynamic properties, molecular mobility, and microbial growth of glucose/WPI composite solid matrices were measured at various *a*_*w*_ and 30 °C. Although the sorption isotherms, *T*_*g*_, and relaxation processes of studied matrices were affected by *a*_*w*_ and WPI, the microbial growth showed highly dependent on water mobility rather than *a*_*w*_. The mobility difference between the system-involved water and liquid pure water was explicated on the basis of a classical thermodynamic viewpoint, and water usability (*U*_*w*_) was introduced to describe the dynamic changes of water mobility in glucose/WPI matrices. Despite to *a*_*w*_, the microbial growth rate was enhanced at high *U*_*w*_ matrices concomitantly with the rapid cell doubling time. Therefore, the proposed *U*_*w*_ parameter, using the information of the dynamic changes of water mobility in a systematic manner, could quantify the water relationship of microorganisms in solid foods as well as have a practical meaning for modulating microbial growth of resultant products. Further research to assess the influence of *U*_*w*_ on growth parameters (i.e., lag phase) and metabolic activity of mold and bacteria will be continually investigated in low-moisture food and pharmaceutical materials.

## Methods

### Food model preparation

α-*D*-Glucose (Crystals; Sigma-Aldrich, St. Louis, USA) and WPI (Powder; carbohydrates or lipids as impurities <10%; Mullins Whey Inc., Mosinee, USA) were used to composite the food model. The glucose and WPI solutions (20%, w/w) were prepared separately in deionized water and subsequently mixed to obtain solutions at different mass ratios (7:3, 1:1, 3:7, and 0:1; w/w). Further, the 5 mL of prepared solutions, loaded in pre-weighed 20 mL glass vials (semi-closed), were frozen in a still-air freezer (DW-HL240, Zhongkemeiling Co., Ltd., China) at −20 °C for 24 h, and then, were subsequently tempered at −80 °C for 3 h prior to lyophilization. The amorphous samples were obtained until the chamber pressure in a laboratory-used freeze dryer (10N/B, Scientz, Ningbo, China) was below 2 bar. It should note that lyophilizing glucose is extremely difficult due to its low *T*_*g*_ and high solubility nature^23^. In this study, therefore, the amorphous glucose was obtained experimentally via a modified quench-cooling approach reported by Simperler and others^29^, in which approximately 1 g of glucose crystals was cooled to −30 °C and melted at 160 °C, and then, quench-cooled again to −30 °C. Three units of each amorphous sample were stored in vacuum desiccators over desiccant (P_2_O_5_; Sigma-Aldrich, St. Louis, USA) to avoid water sorption and reach equilibrium at 30 °C for further analysis.

### Water sorption testing

The amorphous samples were weighed to monitor water sorption behavior as a function of time (24 h intervals until 120 h) over saturated solutions of LiCl, CH_3_COOK, MgCl_2_, K_2_CO_3_, Mg(NO_3_)_2_, NaNO_2_, and NaCl (Sigma-Aldrich, St. Louis, MO, USA) at respective *a*_*w*_ of 0.11, 0.20, 0.31, 0.43, 0.53, 0.65, and 0.75 at storage temperature of 30 °C, in vacuum desiccators. The Guggenheim–Anderson–de Boer (GAB) model (Eq. 7) was applied to fit the water sorption data of each sample, where the *m* and *m*_0_ referred to the weighted water content and monolayer water content; *C*_GAB_ and *K*_GAB_ were constants^21^.

7 $$\frac{m}{{m}_{0}}=\frac{{C}_{{{\rm{GAB}}}}{K}_{{{\rm{GAB}}}}{a}_{w}}{\left(1-{K}_{{{\rm{GAB}}}}{a}_{w}\right)\left(1-{K}_{{{\rm{GAB}}}}{a}_{w}+{{Cka}}_{w}\right)}$$

### Thermal analysis

The thermal properties, including the onset-*T*_*g*_ value for each sample, were determined using a differential scanning calorimeter (DSC; Mettler-Toledo, Schwerzenbach, Switzerland). About 15 mg of prepared samples were transferred into a pre-weighed 50 mL aluminum pan and hermetically sealed before measurement. An empty punctured pan was used as a reference to minimize the systematic error caused by water vapor. Samples were scanned from −20 °C to over the *T*_*g*_ region at 5 °C/min and then cooled at 10 °C/min to the initial temperature. A second heating scan was run well above the *T*_*g*_ at 5 °C/min. The onset-*T*_*g*_ derived from second heating scans were recorded using STARe software (Version 8.10, Mettler-Toledo, Schwerzenbach, Switzerland). The Gordon–Taylor (GT) equation (Eq. 8) had proven to fit experimental onset-*T*_*g*_ data of glucose/WPI solid matrices, where *w*_1_ and *w*_2_ were the mass fractions of amorphous sample and water, *T*_*g*1_ and *T*_*g*2_ were their values, and *k*_*GT*_ was a constant and its thermodynamic meaning discussed later.

8 $$\frac{{W}_{2}}{{T}_{g}-{T}_{g2}}={k}_{{GT}}{w}_{1}\left({T}_{g}-{T}_{g1}\right)$$

### Dynamic-mechanical analysis

The mechanical properties of prepared samples were studied by using a dynamic-mechanical analyzer (DMA; Mettler-Toledo, Schwerzenbach, Switzerland). The loss modulus (*E*″) of materials as a function of temperature at different frequencies (0.5, 1, 3, 5, and 10 Hz) were determined in this study. Before starting an experiment, the instrument was balanced and set at zero to determine the zero-displacement position and return the force to the zero position. Approximately 100 mg samples of ground materials were spread on a titanium pocket-forming sheet. The length, width, and thickness (~2 mm) of the sample pocket between the clamps were measured. Samples were scanned from −20 °C to over the *T*_*g*_ region with a cooling rate of 5 °C/min and a heating rate of 2 °C/min using the single cantilever bending mode to obtain *E*″ values using DMA software (Version 1.43.00, Mettler-Toledo Schwerzenbach, Switzerland). During heating, the samples were analyzed for *T*_*α*_ values determined from the peak temperature of *E*″^28^.

### Molecular mobility determination

The temperature difference (*T*_*α*_ − *T*_*g*_), at which relaxation times (*τ*) exceed time factors critical to the characteristics of the materials, was used to calculate *S* values, which can represent the extent of molecular mobility as noted above. The *τ* and the temperature of *T*_*α*_ above *T*_*g*_ were modeled and analyzed using the WLF equation (Eq. 9), where *T*, *T*_*g*_, *τ*, *τ*_*g*_, refers to the experiment temperature, onset-*T*_*g*_, experimental *a*-relaxation time (oscillation frequency set in DMA measurement, *τ* = ½*πf*), and relaxation time in glass state (≈100 s). The WLF model constants *C*_1_ and *C*_2_ can be derived from a plot of 1/log(*τ*/*τ*_*g*_) against 1/(*T*_*α*_ − *T*_*g*_) using experimental *τ* with the assumption of *τ*_*g*_ = 100 s at the onset-*T*_*g*_^20,35^. Moreover, the *S* value of the system is determined by Eq. (10), where *C*_1_ and *C*_2_ refers to the material-special WLF constants. The Deborah number refers a decrease in the number of logarithmic decades for flow, e.g., to result in stickiness, can be defined as the critical parameter (*d*_*s*_) and a corresponding *T–T*_*g*_ is given as the strength of the solids, *S* parameter. It should be noted that the *S* parameter of carbohydrates-polymeric food systems could be calculated at *d*_*s*_ = 4^36^.

9 $${\rm{Log}}\left(\frac{\tau }{{\tau }_{g}}\right)=\frac{-{C}_{1}\left(T-{T}_{g}\right)}{{C}_{2}+\left(T-{T}_{g}\right)}$$

10 $$S=\frac{{d}_{s}{C}_{2}}{-{C}_{1}-{d}_{s}}$$

Previous studies reported that the compositional dependent of *S* in non-crystalline sugar/protein solids could be represented by Eq. (11). In Eq. (11), *w*_1_ and *w*_2_ referred to the mass fractions of dry solids and water, *k*_*sp*_ was a partition constant of molecular mobility, *S*_*d*1_ and *S*_*d*2_ represented the *S* value for anhydrous solids and amorphous water^37^.

11 $${S}_{p}=\frac{{w}_{1}{S}_{d1}+{k}_{{sp}}{w}_{2}{S}_{d2}}{{w}_{1}+{k}_{s}{w}_{2}}$$

### Microbial response determination

#### Yeast activation

The *D. hansenii* (ACCC 20010; Xuanya Biotechnology Co. Ltd., China), isolated from the natural microflora, was chosen as a targeting microorganism because of its xerotolerant nature. *D. hansenii* was activated prior to inoculation on the bases of the method reported by Sharma and others^34^. The lyophilized strains were dissolved and inoculated in a glassy tube containing 0.5 mL liquid *YM* agar (2.0% glucose, 0.5% yeast extract, 1.0% NaCl, 0.23% NaH_2_PO_4_, 0.5% (NH_4_)_2_SO_4_, and 1.8% agar; Sartorius Stedim Biotech, Globaltec Corp., Germany) at 30 °C on a rotational shaker (200 rpm) for 24 h, and the successful activation achieved when the single colony was obtained.

#### Sample inoculation

The lyophilized sample were stored in vacuum desiccators over P_2_O_5_ as a desiccant to avoid water sorption and used UV light was for 24 h to eliminate environmental effects prior to inoculation. Since the freeze-drying could cause damage to the membrane, DNA, and other cellular components in yeast’s cells, in this study, a tiny quantity of yeast-containing solution (~0.2 μl) was streaked on glucose/WPI solid matrices at mass rations of 1:0, 7:3, 1:1, 3:7, and 0:1. The inoculated glucose/WPI solid matrices were rehumidified over a saturated solution of NaCl, KCl, and K_2_SO_4_ (Sigma-Aldrich, St. Louis, MO, USA) at respective *a*_*w*_ of 0.75, 0.83, and 0.92 *a*_*w*_ at 30 °C, respectively. Previous studies have verified that the low proportion of water incorporated with the inoculation did not raise the water content of the sample at *a*_*w*_ > 0.75^8^. Other equilibrated samples were not inoculated, but rather placed in a closed container over studied storage *a*_*w*_ ranges and temperature for blank control. It is important to know that the whole inoculation was implemented in a clean bench, which can maintain a sterilized condition to avoid contamination from the surrounding ambient.

#### Growth characterization

Scanning electron microscopy (SEM; Phenom Pro, Phenom World. BV, Holland) was used to observe the morphology of microbes in glucose/WPI solid matrices at an acceleration voltage of 10 KV. The studied samples were coated using a gold-palladium alloy coater (Baltec Co., Manchester, NH) and observed at ×8000 magnification. Built-in instrument software (SEM Center, JEOL, Japan) was used for image collection. The growth characters of *D. hansenii* in the glucose/WPI solid matrices were determined by an ATP fluorescence detector (*Pi-102*, Hygiena, USA) at 3 h intervals for 36 h and plotted the growth curve thereafter. The specific growth rate value of the *D. hansenii* in each system was determined by Eq. (12), and the cell doubling time of each system could be determined by Eq. (13). In Eqs. (12) and (13), *μ* was the growth rate (h^−1^), *g* was the cell doubling time (h), *N*_0_ was the number of microbial cells at the beginning, *N*_*t*_ was the number of microbial cells at any time, and *t* was time (h).

12 $$\mu =\frac{(\mathrm{ln}{N}_{t}-\mathrm{ln}{N}_{0})}{t}$$

13 $$g=\frac{\mathrm{ln}(2)}{\mu }$$

### Statistical analysis

The GAB isotherms and *GT* equation, *S* parameter, and microbial growth characteristics of triplicate measurements were analyzed and plotted in Microsoft Excel (2019, Microsoft, Inc., USA). The average values with a standard deviation of triplicate measurements were calculated. In addition, the error bars and significance analysis were implemented in two-sided t-test with the confidence interval of 95% to represent the variability of data.

### Reporting summary

Further information on research design is available in the Nature Research Reporting Summary linked to this article.

## Supplementary information

Supplementary table 1

Reporting Summary

## Data Availability

The datasets generated during and/or analyzed during the current study are available from the corresponding author on request. All data generated or analyzed during this study are included in this paper and its supplementary information files.

## Code availability

The authors declare no custom code or mathematical algorithm is deemed central to the conclusions in this study.

## References

[1] Panitsa A, Petsi T, Kandylis P, Kanellaki M, Koutinas AA (2021). Tubular cellulose from orange juice by-products as carrier of chemical preservatives; delivery kinetics and microbial stability of orange juice. Foods.

[2] Hameed S, Xie L, Ying Y (2018). Conventional and emerging detection techniques for pathogenic bacteria in food science: a review. Trends Food Sci. Technol..

[3] Dao H (2018). Microbial stability of pharmaceutical and cosmetic products. AAPS Pharmscitech.

[4] Havelaar AH (2010). Future challenges to microbial food safety. Int. J. Food Microbiol..

[5] Li X, You B, Shum HC, Chen CH (2022). Future foods: design, fabrication and production through microfluidics. Biomaterials.

[6] Tapia, M. S., Alzamora, S. M., & Chirife, J. in *Water Activity in Foods: Fundamentals and Applications* 323–355 (Blackwell Publishing, 2020).

[7] Chitrakar B, Zhang M, Adhikari B (2019). Dehydrated foods: are they microbiologically safe?. Crit. Rev. Food Sci..

[8] Buera MP, Jouppila K, Roos YH, Chirife J (1998). Differential scanning calorimetry glass transition temperatures of white bread and mold growth in the putative glassy state. Cereal Chem..

[9] Beuchat LR (2013). Low-water activity foods: increased concern as vehicles of foodborne pathogens. J. Food Prot..

[10] Igo MJ, Schaffner DW (2021). Models for factors influencing pathogen survival in low water activity foods from literature data are highly significant but show large unexplained variance. Food Microbiol..

[11] Slade L, Levine H, Reid DS (1991). Beyond water activity: recent advances based on an alternative approach to the assessment of food quality and safety. Crit. Rev. Food Sci. Nutr..

[12] Roos YH (2010). Glass transition temperature and its relevance in food processing. Annu. Rev. Food Sci. Technol..

[13] Stewart CM (2002). Staphylococcus aureus growth boundaries: moving towards mechanistic predictive models based on solute-specific effects. Appl. Environ. Microbiol..

[14] Chirife, J., Buera, M. P., & Gonzalez, H. L. in *Water Management in the Design and Distribution of Quality Foods* 285–298 (CRC Press, 1999).

[15] Lavoie JP, Labbe RG, Chinachoti P (1997). Growth of Staphylococcus aureus as related to ^17^O NMR water mobility and water activity. J. Food Sci..

[16] Kou Y, Molitor PF, Schmidt SJ (1999). Mobility and stability characterization of model food systems using NMR, DSC and conidia germination techniques. J. Food Sci..

[17] Schmidt, S. J. in *Water Activity in Foods: Fundamentals and Applications* 61–122 (Blackwell Publishing, 2020).

[18] Li R, Lin D, Roos YH, Miao S (2019). Glass transition, structural relaxation and stability of spray-dried amorphous food solids: a review. Dry. Technol..

[19] Maidannyk VA, Roos YH (2017). Water sorption, glass transition and “strength” of lactose–Whey protein systems. Food Hydrocoll..

[20] Fan F, Roos YH (2016). Structural relaxations of amorphous lactose and lactose-whey protein mixtures. J. Food Eng..

[21] Cui T, Wu Y, Fan F (2022). Physicochemical properties and Strength analysis of vitreous encapsulated solids for the safe delivery of β-Carotene. Food Res. Int..

[22] Fan F, Cui T, Wu Y (2021). Temporal-spatial property of small molecular sugars and its applications in the processing and storage of sugar-rich foods: a review. Sci. Technol. Food Ind..

[23] Makarem N, Bandera EV, Nicholson JM, Parekh N (2018). Consumption of sugars, sugary foods, and sugary beverages in relation to cancer risk: a systematic review of longitudinal studies. Annu. Rev. Nutr..

[24] Minj S, Anand S (2020). Whey proteins and its derivatives: bioactivity, functionality, and current applications. Dairy.

[25] Hoobin P (2013). Water sorption properties, molecular mobility and probiotic survival in freeze dried protein–carbohydrate matrices. Food Funct..

[26] Fan F, Xiang P, Zhao L (2021). Vibrational spectra analysis of amorphous lactose in structural transformation: Water/temperature plasticization, crystal formation, and molecular mobility. Food Chem..

[27] Fan F, Roos YH (2015). X-ray diffraction analysis of lactose crystallization in freeze-dried lactose–whey protein systems. Food Res. Int..

[28] Potes N, Kerry JP, Roos YH (2012). Additivity of water sorption, alpha-relaxations and crystallization inhibition in lactose–maltodextrin systems. Carbohydr. Polym..

[29] Simperler A (2006). Glass transition temperature of glucose, sucrose, and trehalose: an experimental and in silico study. J. Phys. Chem. B..

[30] Couchman PR, Karasz FE (1978). A classical thermodynamic discussion of the effect of composition on glass-transition temperatures. Macromolecules.

[31] Zhou D, Zhang GG, Law D, Grant DJ, Schmitt EA (2002). Physical stability of amorphous pharmaceuticals: importance of configurational thermodynamic quantities and molecular mobility. J. Pharm. Sci..

[32] Ding S, Peng B, Li Y, Yang J (2019). Evaluation of specific volume, texture, thermal features, water mobility, and inhibitory effect of staling in wheat bread affected by maltitol. Food Chem..

[33] Vittadini E, Dickinson LC, Chinachoti P (2002). NMR water mobility in xanthan and locust bean gum mixtures: possible explanation of microbial response. Carbohydr. Polym..

[34] Sharma P, Meena N, Aggarwal M, Mondal AK (2005). Debaryomyces hansenii, a highly osmo-tolerant and halo-tolerant yeast, maintains activated Dhog1p in the cytoplasm during its growth under severe osmotic stress. Curr. Genet..

[35] Angell CA (1995). Formation of glasses from liquids and biopolymers. Science.

[36] Roos YH (2016). Food engineering at multiple scales: case studies, challenges and the future—a European perspective. Food Eng. Rev..

[37] Wu Y, Huang W, Cui T, Fan F (2021). Crystallization and strength analysis of amorphous maltose and maltose/whey protein isolate mixtures. J. Sci. Food Agric..

