# Supplementary material for: Water usability as a descriptive parameter of thermodynamic properties and water mobility in food solids
Source: NPJ Sci Food. 2023 Jun 14;7:30. doi: 10.1038/s41538-023-00207-0 (PMC10267132; doi:10.1038/s41538-023-00207-0)
Supplement: Supplementary file 1 — Supplementary table 1 [file 41538_2023_207_MOESM1_ESM.pdf]

Supplementary table 1. The a_w, experimental water content, and calorimetric onset-T_g for of noncrystalline glucose and glucose/*WPI* solid matrices (7:3, 1:1 and 3:7; *w/w*) after storage at various water activities (dry ~ 0.4 a_w) and 30 ºC

a_w	Noncrystalline Glucose	T_g (°C)	Glucose/WPI 7:3	T_g (°C)	Glucose/WPI 1:1	T_g (°C)	Glucose/WPI 3:7	T_g (°C)
Dry	Dry	38.2±1.1	Dry	56.5±2.4^{a}	Dry	70.9±2.2^{a}	Dry	93.2±1.2^{a}
0.11±0.01*	0.28±0.05^{a***}	34.6±3.2^{a}	0.35±0.05^{a}	45.4±3.4^{b}	1.51±0.17^{a}	52.6±1.5^{b}	2.29±0.12^{a}	83.1±2.1^{b}
0.20±0.03**	0.61±0.26^{b***}	30.8±4.4^{b}	0.96±0.15^{b}	33.2±3.1^{c}	2.99±0.26^{b}	44.0±2.8^{c}	3.33±0.15^{b}	59.1±4.1^{c}
0.31±0.01	1.36±0.21^{c***}	25.5±2.8^{c}	2.31±0.35^{c}	24.9±2.3^{d}	4.98±0.15^{c}	34.2±3.0^{d}	5.12±0.12^{c}	50.1±2.0^{d}
0.42±0.02	2.52±0.28^{d***}	12.4±4.2^{d}	4.88±0.24^{d}	16.5±3.6^{e}	6.45±0.21^{d}	22.1±4.3^{e}	7.61±0.16^{d}	33.8±3.0^{e}

*: Significant analysis at two-sided t-test, $p \leq 0.05$

**: Values are means ± *SDs* (n = 3)

***: The prediction values
