# Supplementary material for: Water usability as a descriptive parameter of thermodynamic properties and water mobility in food solids
Source: NPJ Sci Food. 2023 Jun 14;7:30. doi: 10.1038/s41538-023-00207-0 (PMC10267132; doi:10.1038/s41538-023-00207-0)
Supplement: Supplementary file 2 — Reporting Summary [file 41538_2023_207_MOESM2_ESM.pdf]

nature portfolio

Corresponding author(s): Fanghui Fan; Tian Mou

Last updated by author(s): May 29, 2023

Reporting Summary

Nature Portfolio wishes to improve the reproducibility of the work that we publish. This form provides structure for consistency and transparency in reporting. For further information on Nature Portfolio policies, see our Editorial Policies and the Editorial Policy Checklist.

Statistics

For all statistical analyses, confirm that the following items are present in the figure legend, table legend, main text, or Methods section.

n/a	Confirmed	
☐	☒	The exact sample size (n) for each experimental group/condition, given as a discrete number and unit of measurement
☐	☒	A statement on whether measurements were taken from distinct samples or whether the same sample was measured repeatedly
☐	☒	The statistical test(s) used AND whether they are one- or two-sided<br>*Only common tests should be described solely by name; describe more complex techniques in the Methods section.*
☒	☐	A description of all covariates tested
☒	☐	A description of any assumptions or corrections, such as tests of normality and adjustment for multiple comparisons
☐	☒	A full description of the statistical parameters including central tendency (e.g. means) or other basic estimates (e.g. regression coefficient) AND variation (e.g. standard deviation) or associated estimates of uncertainty (e.g. confidence intervals)
☒	☐	For null hypothesis testing, the test statistic (e.g. F, t, r) with confidence intervals, effect sizes, degrees of freedom and P value noted<br>*Give P values as exact values whenever suitable.*
☒	☐	For Bayesian analysis, information on the choice of priors and Markov chain Monte Carlo settings
☒	☐	For hierarchical and complex designs, identification of the appropriate level for tests and full reporting of outcomes
☒	☐	Estimates of effect sizes (e.g. Cohen's d, Pearson's r), indicating how they were calculated

Our web collection on statistics for biologists contains articles on many of the points above.

Software and code

Policy information about availability of computer code

Data collection	STARe software (Version 8.10, Mettler-Toledo, Schwerzenbach, Switzerland); Microsoft Excel (version: 2019, Microsoft, Inc., USA)
Data analysis	Microsoft Excel (version: 2019, Microsoft, Inc., USA)

For manuscripts utilizing custom algorithms or software that are central to the research but not yet described in published literature, software must be made available to editors and reviewers. We strongly encourage code deposition in a community repository (e.g. GitHub). See the Nature Portfolio guidelines for submitting code & software for further information.

Data

Policy information about availability of data

All manuscripts must include a data availability statement. This statement should provide the following information, where applicable:

- Accession codes, unique identifiers, or web links for publicly available datasets
- A description of any restrictions on data availability
- For clinical datasets or third party data, please ensure that the statement adheres to our policy

Provide your data availability statement here.

Research involving human participants, their data, or biological material

Policy information about studies with human participants or human data. See also policy information about sex, gender (identity/presentation), and sexual orientation and race, ethnicity and racism.

Reporting on sex and gender	No human participants or human data included in the present study.
Reporting on race, ethnicity, or other socially relevant groupings	No human participants or human data included in the present study.
Population characteristics	No human participants or human data included in the present study.
Recruitment	No human participants or human data included in the present study.
Ethics oversight	No human participants or human data included in the present study.

Note that full information on the approval of the study protocol must also be provided in the manuscript.

Field-specific reporting

Please select the one below that is the best fit for your research. If you are not sure, read the appropriate sections before making your selection.

☒ Life sciences ☐ Behavioural & social sciences ☐ Ecological, evolutionary & environmental sciences

For a reference copy of the document with all sections, see nature.com/documents/nr-reporting-summary-flat.pdf

Life sciences study design

All studies must disclose on these points even when the disclosure is negative.

Sample size	In the present study, no sam0le-size calculation was performed. Instead, the sample size was chosen based on our previous publications and those of others, along with the available resources. All related publications can be found in the references sections of this manuscript.Previous studies with similar sample sizes have successfully achieved their study objectives, which leads us to believe that this sample size is appropriate for our current study. Additionally, we ensured that the sample size is sufficiently large to detect meaningful differences between groups or associations between variables.
Data exclusions	We confirmed that no data were excluded from the analyses in the present study.
Replication	We confirmed that the measures were successfully taken to verify the reproducibility of the experimental findings in the present study.
Randomization	We confirmed that all samples were allocated into experimental groups in randomly.
Blinding	We confirmed that the investigator were blinded to group allocation during data collection and analysis in the present study.

Reporting for specific materials, systems and methods

We require information from authors about some types of materials, experimental systems and methods used in many studies. Here, indicate whether each material, system or method listed is relevant to your study. If you are not sure if a list item applies to your research, read the appropriate section before selecting a response.

Materials & experimental systems

n/a	Involved in the study	
☒	☐	Antibodies
☒	☐	Eukaryotic cell lines
☒	☐	Palaeontology and archaeology
☒	☐	Animals and other organisms
☒	☐	Clinical data
☒	☐	Dual use research of concern
☒	☐	Plants

Methods

n/a	Involved in the study	
☒	☐	ChIP-seq
☒	☐	Flow cytometry
☒	☐	MRI-based neuroimaging
